# Evaluation of positions of four lingula shapes for mandibular ramus surgery

**DOI:** 10.3389/froh.2024.1521227

**Published:** 2025-01-07

**Authors:** Kun-Jung Hsu, Pei-Jung Chen, Han-Sheng Chen, Kun-Tsung Lee, Chun-Ming Chen

**Affiliations:** ^1^ School of Dentistry, College of Dental Medicine, Kaohsiung Medical University, Kaohsiung, Taiwan; ^2^ Department of Dentistry, Kaohsiung Medical University Hospital, Kaohsiung, Taiwan; ^3^ Dental Department, Kaohsiung Municipal Siao-Gang Hospital, Kaohsiung, Taiwan; ^4^ Department of Oral Hygiene, College of Dental Science, Kaohsiung Medical University, Kaohsiung, Taiwan; ^5^ Department of Oral and Maxillofacial Surgery, Kaohsiung Medical University Hospital, Kaohsiung Medical University, Kaohsiung, Taiwan

**Keywords:** cone beam computerized tomography, mandibular foramen, lingula shape, sagittal split ramus osteotomy, intraoral vertical ramus osteotomy

## Abstract

**Background:**

The lingula is an important landmark for conducting certain mandibular surgery procedures, such as sagittal split ramus osteotomy (SSRO) and intraoral vertical ramus osteotomy (IVRO). The purpose of this study was to investigate the location of the lingula in both horizontal and vertical planes among four different shapes of the mandibular ramus.

**Methods:**

Ninety patients, 60 female and 30 male, underwent cone beam computed tomography scans to evaluate the measurements of the lingula tip (Li) in relation to the anterior border (AB), posterior border (PB), sigmoid notch (SN), and inferior border (IB) of the ramus. The proportional relationship of the Li in both the horizontal and vertical planes is indicated by the Li–AB/AB–PB ratio and Li–SN/SN–IB. lingula shapes were categorized into triangular, truncated, nodular, and assimilated shapes. Statistical analyses were performed to compare the variations in the measurements between different shapes of lingula and genders.

**Results:**

The mean Li–AB distance was 18.88 mm, and it was determined to be significantly greater with the truncated shape when compared to the other three shapes. The mean Li–PB distance was 15.23 mm, with no significant differences observed among the four shapes. The mean Li–AB/AB–PB ratio was found to be 55.3%. The truncated shape had a ratio of 57.2%, which was significantly higher compared to the nodular shape (54%) and assimilated shape (50.4%). The mean distance was 19.95 mm for the Li–SN and 31.34 mm for the Li–IB. There was no significant difference in these distances among the 4 lingula shapes. The mean Li–SN/SN–IB ratio was 38.5%. There were no significant differences in measurements between male and female.

**Conclusion:**

Significant differences were observed in the 4 lingula shapes in relation to the Li position, which was situated superiorly and posteriorly to the central point of the ramus. Therefore, it is crucial to take into account the differences in lingula shapes during SSRO and IVRO procedures on the ramus.

## Background

The mandibular lingula is a prominent bony ridge found on the medial side of the mandible. It is positioned above the mandibular foramen and features a tongue-like shape. Tuli et al. (1) classified the lingula into four different shapes: triangular, truncated, nodular, and assimilated. The sphenomandibular ligament (SML) is a tough, flat, and thin fibrous band originating from Meckel's cartilage (2). The superior portion of the SML attaches to the spine of the sphenoid bone, while the inferior part of the SML attaches to the lingula and the lower border of the mandibular foramen (MF). The MF allows for the entry of the inferior alveolar nerve (IAN) and blood vessels into the mandibular canal. It is crucial to understand the position of the lingula in relation to the mandibular foramen (MF) and inferior alveolar nerve (IAN). This knowledge can enhance the success and efficiency of IAN blocks during dental anesthesia, ultimately improving the effectiveness of dental procedures and related surgical interventions.

Hsu et al. (3) performed a literature review examining the morphological traits of the mandibular lingula. Their analysis revealed that in Indian populations, the triangular shape was the most commonly observed among both male and female. Conversely, the truncated shape was more frequently found in both genders within Thai and Brazilian populations. When considering the source of the mandibles—dry specimens vs. cone beam computed tomography (CBCT)—the most frequently observed lingula type in dry mandibles was also triangular, followed by truncated, nodular, and assimilated forms. In the CBCT group, however, the nodular type was the most commonly observed, followed by truncated, triangular, and assimilated shapes. The differences noted in the dry mandible cohort may be influenced by various factors including age, ethnicity, dental condition, and skeletal characteristics of the samples, in addition to the methodologies employed for the collection and preservation of human mandibles. In contrast, while CBCT images are unaffected by the preparation and preservation processes of dry mandibles, the researchers' interpretation of these images may be influenced by potential reductions in image clarity that can occur during software processing.

In the surgical management of mandibular prognathism, there are two main surgical techniques used for mandibular setback procedures: sagittal split ramus osteotomy (SSRO) and intraoral vertical ramus osteotomy (IVRO). The two techniques vary in that: (1) During the SSRO procedure, the surgeon employs specialized instruments to carefully dissect the medial soft tissue above the mandibular foramen to protect the inferior neurovascular buddle. Subsequently, the ramus is meticulously split into medial and lateral bone segments. (2) During the IVRO procedure, the osteotomy is precisely performed behind the lingula and MF from the lateral aspect of the ramus. Subsequently, the ramus is divided into distal and proximal segments. The positions of the lingula and the mandibular foramen are crucial for selecting the appropriate surgical approach. A thorough understanding of these anatomical locations is essential to minimizing the risk of injury to the IAN and adjacent blood vessels during the procedure, as damage to these structures could result in postoperative numbness in the lower lip (4). Therefore, it is essential to carefully assess the shape of the lingula and accurately measure the related distances in order to effectively carry out SSRO and IVRO procedures. The purpose of this study was to assess the horizontal and vertical dimensions of four different shapes of the lingula in the ramus, and to identify any significant differences among these four shapes.

## Methods

**Figure 1:**
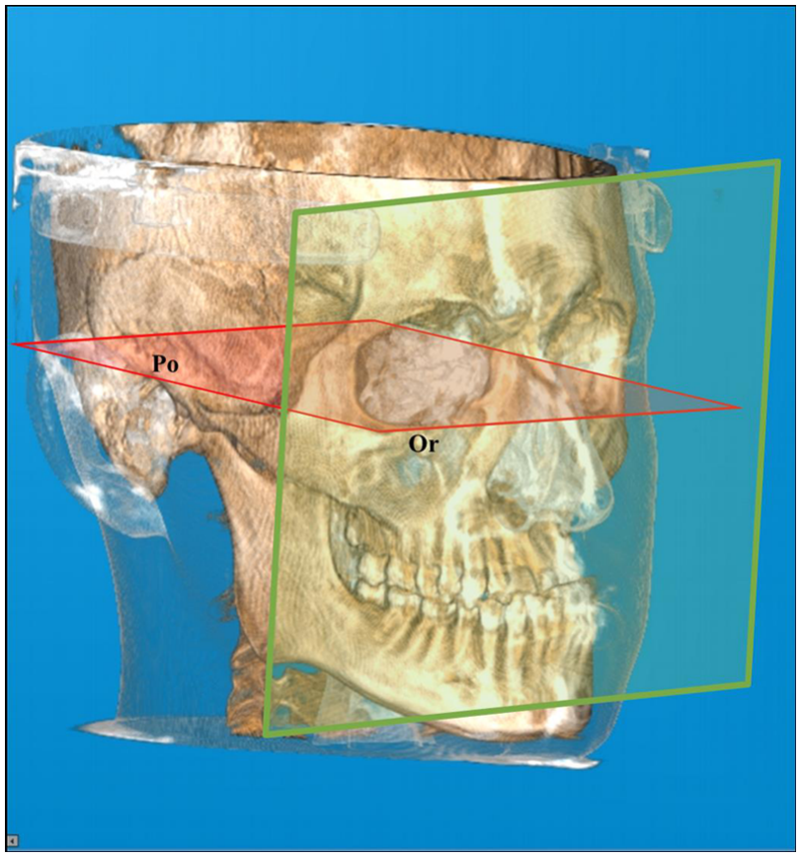
The frankfort horizontal plane (red): a horizontal reference plane extending through the inferior rim of the orbital cavity (Or: orbitale) and the superior part of the external auditory meatus (Po: porion). Vertical plane (green): a vertical reference plane perpendicular to the Frankfort horizontal plane.

**Figure 2:**
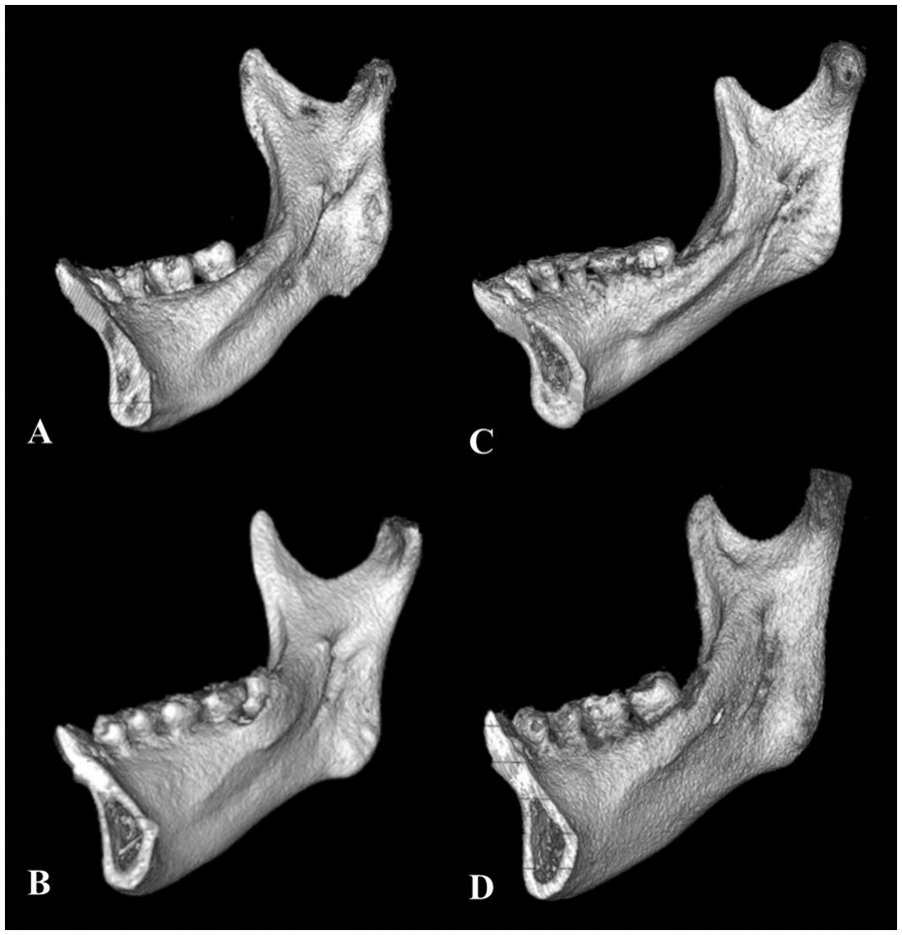
Four shapes of lingula were classified. **(A)** Triangular, **(B)** Truncated, **(C)** Nodular, and **(D)** Assimilated.

**Figure 3:**
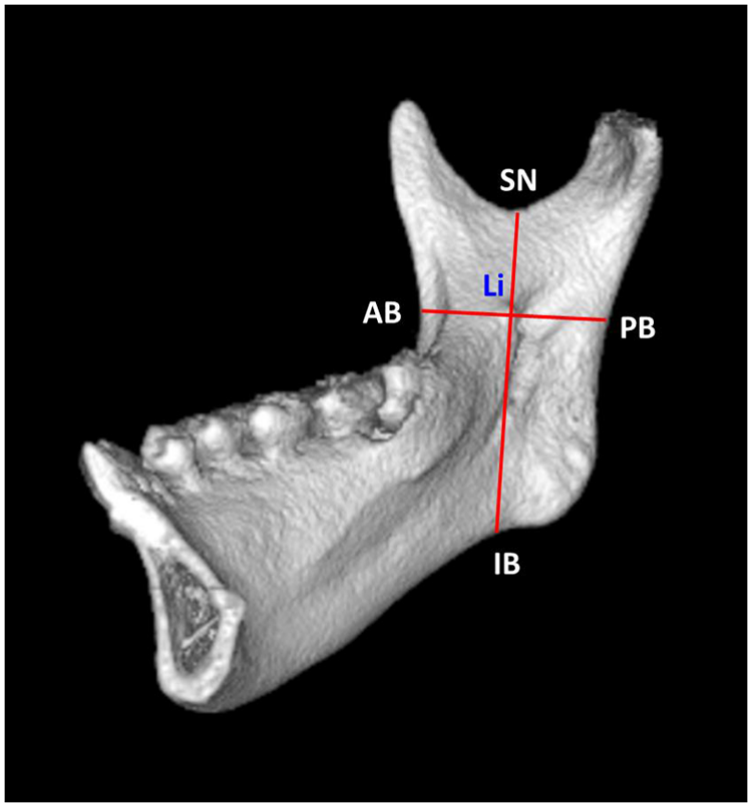
Distance measurements and landmarks in the vertical reference plane and horizontal reference plane. Tip of the lingula (Li), anterior border of the ramus (AB), posterior border of the ramus (PB), sigmoid notch (SN), and inferior border of the ramus (IB).

The patient underwent CBCT imaging at Kaohsiung Medical University Chung-Ho Memorial Hospital's Density Department. The images were captured while the patient maintained a natural head position and central occlusion. Patients were excluded if they had any of the following conditions: (1) craniofacial tumors or pathologies, (2) congenital craniofacial deformities, or (3) a history of craniofacial trauma or surgery. The CBCT DICOM files were imported into RadiAnt DICOM Viewer version 4.6.9 (Medixant, Poznan, Poland) to create three-dimensional images. The software's built-in measurement tools, including a ruler, were utilized to assess the relevant distances. The horizontal reference plane (Figure 1) for the three-dimensional images, known as the Frankfort horizontal plane, extending through inferior rim of the orbital cavity (Or: orbitale) and the superior part of the external auditory meatus (Po: porion). According to the classification system by Tuli et al., the shapes of the lingula were classified into four categories: triangular, truncated, nodular, and assimilated (Figure 2). In the Figure 3, the measurements were taken using the vertical and horizontal lines that intersected at the tip of the lingula (Li). During the horizontal measurements, the following data was obtained: (1) the Li–AB distance, which is the distance from the Li to the anterior border of the ramus; (2) the Li–PB distance, which is the distance from the Li to the posterior border of the ramus; (3) the AB–PB distance, which is the distance between the anterior and posterior borders of the ramus; and (4) the Li–AB/AB–PB ratio, which indicates the relative position of the Li between the anterior and posterior borders of the ramus. The following vertical measurements were recorded: (1) the Li-SN distance, which is the distance from the Li to the sigmoid notch (SN); (2) the Li-IB distance, which is the distance from the Li to the inferior border of the ramus (IB); (3) the SN-IB distance, which is the height of the ramus measured as the distance from the SN to IB; and (4) the Li-SN/SN-IB ratio, which indicates the relative position of the Li between the SN and IB.

Statistical analysis was conducted using IBM SPSS 20 (SPSS, Chicago, IL, USA) to investigate the distances related to the four different shapes of the lingula. An analysis of variance (ANOVA) was employed to compare the differences among four groups. Subsequently, the Scheffe *post hoc* test was employed to investigate any significant differences found in the ANOVA results. A *p*-value of <0.05 was considered to be statistically significant. Furthermore, this study examined and compared the findings obtained from both male and female participants. The study was approved by the institutional review board of Kaohsiung Medical University (IRB No. KMUH-IRB-20160066).

## Results

In this study, we analyzed the CBCT images from 90 patients, encompassing a total of 180 sides (Table 1). The triangular shape was found in 44 sides, the truncated shape in 59 sides, the nodular shape in 68 sides, and the assimilated shape in 9 sides (5). Among these patients, there were 60 female (120 sides) and 30 male (60 sides). In female patients, the triangular shape was detected in 31 sides, the truncated shape in 37 sides, the nodular shape in 48 sides, and the assimilated shape in 4 sides. In male patients, the triangular shape was observed in 13 sides, the truncated shape in 22 sides, the nodular shape in 20 sides, and the assimilated shape in 5 sides.

**Table 1:** Horizontal distances (mm) and ratios of lingula shapes in the total sides.

	Li-AB		Li-PB		AB-PB		Li-AB/AB-PB
	Mean	SD	Mean	SD	Mean	SD	Ratio
Shape							
Triangular (*n* = 44)	18.64	2.47	14.73	1.92	33.37	2.67	0.558
Truncated (*n* = 59)	20.30	2.59	15.23	2.26	35.53	3.55	0.572
Nodular (*n* = 68)	18.08	2.40	15.39	1.81	33.47	3.09	0.540
Assimilated (*n* = 9)	16.83	1.72	16.57	1.95	33.40	3.27	0.504
Total (*n* = 180)	18.88	2.66	15.23	2.02	34.12	3.29	0.553
Intershape comparison							
*p* vlaue	<0.001*		0.071		0.001*		<0.001*
	2 > 1,3,4		NS		2 > 1,3		2 > 3,4; 1 > 4

*n*, number of side; Li, lingula; AB, anterior border of ramus PB, posterior border of ramus.

Lingula shape: 1, Triangular; 2, Truncated; 3, Nodular; 4, Assimilated.

* Significant, *p* < 0.05; NS: Not Significant.

Among the total participants (Table 1), the average distance between the Li and AB points was 18.88 mm. The average distances for the triangular, truncated, nodular, and assimilated shapes were 18.64, 20.30, 18.08, and 16.83 mm, respectively. It is important to note that the mean Li-AB distance for the truncated shape was significantly larger than that of the other three shapes. The mean Li–PB distance was 15.23 mm, with no significant differences observed among the four shapes. The mean AB–PB distance was 34.12 mm among all participants. Additionally, the truncated shape exhibited significantly greater measurements in comparison to both the triangular and nodular shapes. The mean Li–AB/AB–PB ratio was 55.3% among all participants. The mean Li–AB/AB–PB ratio for the truncated shape was found to be significantly higher at 57.2% compared to the nodular shape at 54% and the assimilated shape at 50.4%.

In the vertical measurements (Table 2), the mean Li–SN distance was 19.59 mm among all participants. The mean distances for the triangular, truncated, nodular, and assimilated shapes were 19.03, 19.97, 19.76, and 18.61 mm, respectively. No statistically significant differences were observed among these four shapes. The mean Li–IB distance was 31.34 mm among all participants, while the mean SN–IB distance was 50.94 mm. Regarding these distances, no statistically significant differences were observed among the four different shapes. The mean Li–AB/AB–PB ratio was 38.5% among all participants. The truncated shape was significantly higher at 39.4% compared to the triangular shape at 37.3% and the assimilated shape at 35.1%. Additionally, the average ratio for the nodular shape was significantly higher at 38.8% compared to the assimilated shape.

**Table 2:** Vertical distances (mm) and ratios of lingula shapes in the total sides .

	Li-SN		Li-IB		SN-IB		Li-SN/SN-IB
	Mean	SD	Mean	SD	Mean	SD	Ratio
Shape							
Triangular (*n* = 44)	19.03	3.54	31.80	3.51	50.83	4.67	0.373
Truncated (*n* = 59)	19.97	2.90	30.76	4.07	50.73	4.88	0.394
Nodular (*n* = 68)	19.76	3.17	31.15	3.88	50.92	5.05	0.388
Assimilated (*n* = 9)	18.61	3.23	34.39	4.25	53.00	6.04	0.351
Total (*n* = 180)	19.59	3.19	31.34	3.92	50.94	4.94	0.385
Intershape comparison							
*p* vlaue	0.357		0.058		0.641		0.026*
	NS		NS		NS		2 > 1,4; 3 > 4

*n*, number of side; Li, lingula; SN, sigmoid notch; IB, inferior border of ramus.

Lingula shape: 1, Triangular; 2, Truncated; 3, Nodular; 4, Assimilated.

* Significant, *p* < 0.05; NS: Not Significant.

In female participants (Tables 3, 4), the mean Li–AB distance for the truncated shape (19.52 mm) was significantly greater than those for the nodular shape (18.12 mm) and assimilated shape (16.15 mm). The mean Li–AB/AB–PB ratio was higher for the truncated shape (56.9%) compared to the nodular shape (54.7%) and assimilated shape (50.1%). Additionally, the mean ratio for the triangular shape (56.1%) was also significantly greater than that for the assimilated shape. There were no significant differences in the mean Li-SN, Li-IB, and SN-IB distances in the vertical direction among the four shapes. The Li-SN/SN-IB ratios were significantly higher in the nodular shape (39.9%) and truncated shape (39.6%) compared to the assimilated shape (34.5%).

**Table 3:** Horizontal distances (mm) and ratios of lingula shapes in the females .

	Li-AB		Li-PB		AB-PB		Li-AB/AB-PB
	Mean	SD	Mean	SD	Mean	SD	Ratio
Shape							
Triangular (*n* = 31)	18.48	2.22	14.45	1.95	32.94	2.54	0.561
Truncated (*n* = 37)	19.52	2.35	14.84	2.26	34.36	3.06	0.569
Nodular (*n* = 48)	18.12	2.40	14.98	1.85	33.10	3.21	0.547
Assimilated (*n* = 4)	16.15	2.34	16.05	2.29	32.20	4.22	0.501
Total (*n* = 120)	18.58	2.43	14.84	2.02	33.42	3.06	0.556
Intershape comparison							
*p* vlaue	0.009*		0.424		0.143		0.019*
	2 > 3,4		NS		NS		2 > 3,4; 1 > 4

*n*, number of side; Li, lingula; AB, anterior border of ramus; PB, posterior border of ramus.

Lingula shape: 1, Triangular; 2, Truncated; 3, Nodular; 4, Assimilated.

* Significant, *p* < 0.05; NS: Not Significant.

**Table 4:** Vertical distances (mm) and ratios of lingula shapes in the females.

	Li-SN		Li-IB		SN-IB		Li-SN/SN-IB
	Mean	SD	Mean	SD	Mean	SD	Ratio
Shape							
Triangular (*n* = 31)	19.14	3.30	31.20	3.44	50.34	4.75	0.379
Truncated (*n* = 37)	19.44	2.73	29.74	3.50	49.19	4.50	0.396
Nodular (*n* = 48)	19.96	3.30	29.96	3.54	49.92	5.26	0.399
Assimilated (*n* = 4)	16.73	1.51	31.68	0.82	48.40	1.53	0.345
Total (*n* = 120)	19.48	3.12	30.27	3.47	49.75	4.80	0.391
Intershape comparison							
*p* vlaue	0.200		0.253		0.722		0.043*
	NS		NS		NS		2 > 4; 3 > 4

*n*, number of side; Li, lingula; SN, sigmoid notch; IB, inferior border of ramus.

Lingula shape: 1, Triangular; 2, Truncated; 3, Nodular; 4, Assimilated.

* Significant, *p* < 0.05; NS: Not Significant.

In male participants (Tables 5, 6) revealed that the mean Li–AB distance for the truncated shape (21.62 mm) was significantly greater than those for the triangular shape (19.02 mm), nodular shape (17.99 mm), and assimilated shape (17.38 mm). The mean AB–PB distance for the truncated shape (37.50 mm) was significantly greater than those for the triangular shape (34.42 mm) and nodular shape (34.35 mm). The mean Li–AB/AB–PB ratio for the truncated shape (57.7%) was significantly greater than those for the triangular shape (55%) and nodular shape (52.2%). In the vertical measurements, no significant differences were observed in the average distances between Li-SN, Li-IB, and SN-IB, nor in the mean ratio of Li-SN to SN-IB among the four shapes. There were no significant differences found between male and female participants regarding the horizontal measurements, vertical measurements, Li–AB/AB–PB ratio, and Li–SN/SN–IB ratio.

**Table 5:** Horizontal distances (mm) and ratios of lingula shapes in the males .

	Li-AB		Li-PB		AB-PB		Li-AB/AB-PB
	Mean	SD	Mean	SD	Mean	SD	Ratio
Shape							
Triangular (*n* = 13)	19.02	3.04	15.40	1.74	34.42	2.79	0.550
Truncated (*n* = 22)	21.62	2.49	15.88	2.16	37.50	3.50	0.577
Nodular (*n* = 20)	17.99	2.45	16.37	1.32	34.35	2.65	0.522
Assimilated (*n* = 5)	17.38	0.99	16.98	1.79	34.36	2.34	0.507
Total (*n* = 60)	19.49	3.00	16.03	1.80	35.52	3.30	0.547
Intershape comparison							
*p* vlaue	<0.001*		0.287		0.004*		0.001*
	2 > 1,3,4		NS		2 > 1,3		2 > 3,4
Intergender comparison							
*p* vlaue	0.521		0.136		0.094		0.540
	NS		NS		NS		NS

*n*, number of side; Li, lingula; AB, anterior border of ramus; PB, posterior border of ramus.

Lingula shape: 1, Triangular; 2, Truncated; 3, Nodular; 4, Assimilated.

* Significant, *p* < 0.05; NS: Not Significant.

**Table 6:** Vertical distances (mm) and ratios of lingula shapes in the males.

	Li-SN		Li-IB		SN-IB		Li-SN/SN-IB
	Mean	SD	Mean	SD	Mean	SD	Ratio
Shape							
Triangular (*n* = 13)	18.77	4.19	33.22	3.37	51.99	4.43	0.359
Truncated (*n* = 22)	20.85	3.02	32.48	4.45	53.33	4.45	0.392
Nodular (*n* = 20)	19.29	2.87	34.02	3.14	53.31	3.59	0.361
Assimilated (*n* = 5)	20.12	3.57	36.56	4.73	56.68	5.76	0.355
Total (*n* = 60)	19.82	3.33	33.49	3.92	53.31	4.34	0.372
Intershape comparison							
*p* vlaue	0.271		0.175		0.241		0.174
	NS		NS		NS		NS
Intergender comparison							
*p* vlaue	0.756		0.080		0.288		0.234
	NS		NS		NS		NS

*n*, number of side; Li, lingula; SN, sigmoid notch; IB, inferior border of ramus.

NS: Not Significant.

## Discussion

Performing surgeries near the lingula, MF, or mandibular canal in the mandibular ramus carries the potential risk of injuring the inferior alveolar neurovascular bundle. This could result in significant bleeding during the operation and numbness in the lower lip after surgery. Hence, accurately identifying the exact positions of the lingula, MF, and occlusal plane (OP), as well as the measurements between them, is crucial during the ramus surgery.

## Distances from anterior and posterior borders of ramus to lingula tip (Li–Ab and Li–Pb distances)

Several studies (6–8) have been carried out on dry mandibles. Jansisyanont et al. (6) reported a mean Li–AB distance of 20.6 mm in a Thai population, and Park et al. (7) reported a mean Li–AB distance of 18.89 mm in a South Korean population. Monnazzi et al. (8) also found that in a Brazilian population, the mean Li–AB distance was 16.50 mm. Numerous studies (9–11) have been conducted using CBCT images of participants. In the Turkish population, Sekerci and Sisman (9) reported a mean Li–AB distance of 16.77 mm, while Senel et al. (10) found a mean Li–AB distance of 18.5 mm. In the Italian population, Lupi et al. (11) reported a mean Li–AB distance of 16.96 mm. In our study, the mean Li–AB distance was 18.88 mm. The differences seen in the studies mentioned indicate that there are variations in results based on ethnicity when using direct measurements from dry mandibles vs. indirect measurements from CBCT. The positioning of the mandible and the various horizontal and vertical reference planes and points utilized in each study significantly impacted the measurement outcomes.

In total participants, the assimilated shape had the shortest mean Li-AB distance of 16.83 mm, while the truncated shape had the longest mean distance of 20.30 mm. The Li-AB distance of truncated shape was significantly longer compared to the other three shapes. Therefore, surgeons are recommended to proceed cautiously when performing a medial horizontal osteotomy in SSRO procedures for patients with a truncated lingula. To ensure reaching the Li, surgeons should consider increasing the length of the osteotomy by 3–4 mm than assimilated shape. In this scenario, it is essential to employ preoperative three-dimensional radiographic imaging. In addition, our study found no statistically significant differences between sexes in the location of the Li among the four lingula shapes.

Investigating the Li–PB distance and Li–AB/AB–PB ratio, Jansisyanont et al. (6) found that the mean Li-PB distance was 18 mm, with a Li-AB/AB-PB ratio of 53.2%. Park et al. (7) reported a mean Li–PB distance of 18.89 mm and a Li-AB/AB-PB ratio of 55%., Monnazzi et al. (8) observed a mean Li-PB distance of 14.63 mm and a Li-AB/AB-PB ratio of 53%. Sekerci and Sisman (9) calculated a mean Li-PB distance of 13.02 mm and a Li-AB/AB-PB ratio of 56%. Senel et al. (10) found a mean Li-PB distance of 16.9 mm and a Li-AB/AB-PB ratio of 53%. Lupi et al. (11) reported a mean Li–PB distance of 15.28 mm and a Li-AB/AB-PB ratio of 53%. In the present study, the mean Li–PB distance was 15.23 mm, and this distance did not significantly vary among the four different lingula shapes. Furthermore, the Li was located at 55% of the distance between the AB and PB. The truncated shape showed the highest Li-AB/AB-PB ratio at 57%, compared to the assimilated shape which only had a ratio of 50%. There were significant differences in the ratios among the four lingula shapes, indicating that the particular shape of the lingula influences the position of Li in the anterior-posterior plane of the mandible.

## Distances from sigmoid notch and inferior border to lingula tip (Sn–Li and Ib–Li distances)

Jansisyanont et al. (6) reported a mean SN–Li distance of 16.6 mm. Lupi et al. (11) *n* found that the mean SN–Li distance of 13.87 mm, with a mean IB–Li distance of 31.2 mm, and the Li positioned at 31% of the SN-IB distance. Alves and Deana (12) reported a mean SN–Li distance of 17.29 mm and a mean IB–Li distance of 33.3 mm, with the Li situated at 34% of the SN-IB distance. Senel et al. (10) found that the mean SN–Li distance of 18.1 mm and the mean IB-Li distance was 38.3 mm, with the Li located at 32% of the SN-IB distance. In present study, the mean SN–Li and IB–Li distance were 19.59 mm and 31.34 mm, respectively. There were no significant differences in both distances observed among the four different lingula shapes. The location of the Li was found to be at 38.5% of the SN-IB distance. Differences among the four shapes were statistically significant. The Li–SN/SN–IB ratios for the nodular shape (39.9%) and truncated shape (39.6%) were significant higher than that of the assimilated shape (34.5%).

Our findings indicated that the morphology of the lingula plays a significant role in determining its position relative to the superior-inferior plane. Previous studies (6, 10–12) have identified variations in the length measurements of the lingula's position influenced by factors such as ethnicity, age, gender, anatomical reference points, and the specific landmarks used in the analysis. However, the ratio of SN–Li to SN-IB appears to show only slight discrepancies when comparing data obtained from dry mandibles with that from CBCT images. In our study, there are no analyses comparing the right and left sides. As a result, we lack information on any potential differences between the two sides for the same individual or across all individuals studied. Moving forward, we aim to assess whether the average distance and ratio are consistent between the right and left sides, or if any differences are present.

During SSRO, it is essential for surgeons to take into account not only the dimensions of the Li and MF but also the height of the lingula. In a study conducted by Alves and Deana (12), it was found that the average lingular height in a Brazilian Caucasian population was 8.89 mm for male and 7 mm for female. Similarly, Zhou et al. (13) reported that the mean lingular height for Korean adults was 10.1 mm for male and 9.8 mm for female, with no significant differences noted between the genders. Additionally, Hsu et al. (14) observed that the average lingular height for male in a Taiwanese population was 8.73 mm, which was significantly higher than the 7.76 mm recorded for female. Furthermore, regarding the position of the lingula in relation to the occlusal plane, Jansisyanont et al. (6) found that 80% of the lingulae were located 4.5 mm above the occlusal plane. Zhou et al. (13) noted that the lingula is rarely positioned below the occlusal plane, with most being found approximately 5.9 ± 3.0 mm above it. Additionally, Akcay et al. (15) reported that the measurement for Class III (mean 9.91 mm) was significantly greater than that for Class I (mean 8.12 mm) within a Turkish population.

Furthermore, it is important to evaluate the thickness of the cortical and cancellous bones between the SN and Li, as well as the area of fusion between the medial and lateral cortices. If the cancellous bone is thin, performing a medial horizontal osteotomy during SSRO to split the medial and lateral bone segments may increase the risk of fractures in the outer cortex of the ramus and result in a bad split. This risk persists even if the osteotomy does not directly engage the outer cortex. Smith et al. (16) found that the distance between the Li and the fusion point along the vertical plane varied between 7.5 and 13.3 mm. They recommended that the medial horizontal osteotomy should be performed at or just above the Li level. They also highlighted that performing the medial horizontal osteotomy at a higher position could lead to increased challenges in bone splitting or a higher risk of unfavorable fractures. Tom et al. (17) found that if a medial horizontal osteotomy is performed more than 5 mm above the Li, there is a higher chance of cutting into the fusion point of the medial and lateral cortices. Suzen et al. (18) explored the impact of medial horizontal osteotomy positioning on postoperative complications and sensory deficits. Their findings indicated that performing the osteotomy above the Li significantly reduced the risk of complications and sensory deficits compared to osteotomies performed below it. Performing an osteotomy in the Li region, where there is adequate cancellous bone width, can minimize the risk of bone split occurring solely within the cortical bone.

Jansisyanont et al. (6) found that the width of MF was measured to be 4.7 mm, with a range of 2.9 to 6.8 mm. Park et al. (7) reported that the posterior margin of the MF to AB and PB were 19.69 mm and 14.41 mm, respectively. The posterior margin of the MF was located at approximately 58% anteriorly and 46% superiorly in the ramus. Apinhasmit et al. (19) reported the posterior margin of the MF to AB and PB were 22.3 mm and 12.7 mm, respectively. The posterior margin of the MF was situated around 64% anteriorly in the ramus. These findings indicate that the posterior margin of the MF is primarily located behind the lingula. It is crucial to consider the positions of the Li and MF when carrying out procedures such as SSRO and IVRO. During SSRO, Wolford (20) advised that the medial horizontal osteotomy should be positioned above the Li and extended towards the area behind the Li and MF. Muto et al. (21) analyzed the distribution of cancellous bone following the application of medial horizontal osteotomy during SSRO. It was observed that individuals with Class III skeletal patterns exhibited a thinner, more irregular, and inconsistent distribution of cancellous bone. This characteristic was particularly evident in the anterior and posterior regions of the MF. Therefore, they determined that the optimal and safest position for a medial horizontal osteotomy is directly above the Li, with an extension slightly posteriorly by 5 to 6 mm.

To perform a successful SSRO procedure, it is important to confirm the location of the lingula relative to the occlusal plane using medical imaging. Careful and meticulous handling of surgical instruments is crucial to avoid accidental injury to the lingula or mandibular foramen. Such damage could lead to significant bleeding during surgery and may cause numbness of the lower lip postoperatively. In medial horizontal osteotomy during SSRO, it is not essential to visually or manually confirm the position of the lingula. To reduce the risk of injuring the IAN and associated vascular structures, we recommend that the osteotomy be conducted at least 5 mm above the occlusal plane.

## Conclusion

Our study revealed notable variations in the locations of the four lingula shapes. Significant differences were observed in the positioning of Li, both along the horizontal and vertical axes, as well as in the ratios of Li-AB/AB-PB and Li-SN/SN-IB. The average Li-AB distance for the truncated shape was significantly greater than that of the other three shapes. Therefore, it is crucial to take into account the variations in the shapes of the lingula and associated distances when conducting SSRO and IVRO procedures on the ramus.

## Data Availability

The original contributions presented in the study are included in the article/Supplementary Material, further inquiries can be directed to the corresponding author/s.
